# Time-of-day dependent promotion of keratinocyte differentiation by *Cinnamomum cassia* bark extract through the p38 MAPK Pathway

**DOI:** 10.1371/journal.pone.0318360

**Published:** 2025-03-18

**Authors:** Miji Yeom, Kyungeun Jeon, De-Hun Ryu, Deokhoon Park, Eunsun Jung

**Affiliations:** Biospectrum, Life Science Institute, Yongin-Si, Republic of Korea; Karlsruhe Institute of Technology: Karlsruher Institut fur Technologie, GERMANY

## Abstract

The skin serves as an essential barrier against pathogens and external insults, preventing moisture loss. Chronic skin conditions such as atopic dermatitis stem from impairments in skin barrier function. Circadian rhythms affect skin blood flow and barrier characteristics, which are significant for understanding atopic dermatitis. *Cinnamomum cassia* bark, commonly known as cinnamon, is extensively utilized in both modern and Traditional Chinese Medicine for its therapeutic properties in managing chronic diseases. This study aimed to investigate the potential use of *Cinnamomum cassia* bark in enhancing skin barrier function. We examined the impact of *Cinnamomum cassia* bark extract (CCBE) on circadian clock-mediated enhancement of the skin barrier. CCBE enhanced the expression of keratinocyte differentiation markers, including keratin 10, filaggrin, caspase 14, and calpain-1. CCBE also increased the production of hyaluronic acid protein. Additionally, CCBE improved the circadian rhythm of period circadian regulator 2 (*PER2*). Notably, CCBE upregulated the expression of keratinocyte differentiation markers and *PER2* specifically during the morning hours. Furthermore, we discovered that siRNA-mediated PER2 knockdown diminished the increase in keratinocyte differentiation markers induced by CCBE. These findings demonstrate that CCBE can regulate the differentiation of keratinocytes in a time-of-day-dependent manner via the circadian clock. CCBE augmented phosphorylation of p38 and JNK, while the CCBE-induced enhancement in *FLG* expression and *PER2* circadian rhythm was reduced by p38 MAPK inhibitors. These results suggest that CCBE can strengthen the skin barrier diurnally via the p38 MAPK pathway, representing a novel and more effective method for enhancing skin barrier function that accommodates daily variations in skin barrier properties.

## 1. Introduction

The skin acts as a protective barrier against pathogens, chemicals, physical agents, and water loss [1,2]. This barrier function is accomplished through epidermal differentiation and cornification, resulting in a “brick and mortar” structure composed of corneocytes embedded in a lipid-rich matrix. This structure provides mechanical flexibility to the skin and inhibits water evaporation [3,4]. Chronic skin conditions such as atopic dermatitis result from abnormalities in epidermal keratinization, which include reduced hydration, elevated trans-epidermal water loss, and diminished sebum production [5–7].

The cornified envelope is formed through the precise differentiation of keratinocytes, identifiable by morphological cell changes or shifts in specific protein expressions. The establishment of calcium gradients instigates these protein changes. Initially, keratin 5 and keratin 14 are supplanted by keratin 1 and keratin 10. Subsequently, involucrin (IVL) and transglutaminase-1 (TGM1) are expressed, followed by late differentiation markers loricrin (LOR) and filaggrin (FLG). FLG precursors can cross-link with keratin to form a corneocyte with a compressed shape. Moreover, TGM1 cross-links IVL, loricrin, and various structural proteins to forge a robust cornified envelope, enhancing the barrier [8–10]. Keratinocytes also produce natural moisturizing factors from the breakdown of proteins such as FLG, which help retain water and shield the skin. FLG is converted into NMF by proteolytic enzymes like calpain-1 (CAPN1) and caspase-14 (CASP14) [11–13].

The MAPK family, which includes pathways such as p38 MAPK, ERK1/2 MAPK, and JNK signaling, plays a pivotal role in keratinocyte differentiation and skin barrier maintenance. These pathways regulate various proteins involved in differentiation and integrate multiple signals critical for keratinocyte differentiation [14–16]. Inhibiting the p38, ERK1/2, and JNK pathways has been shown to decrease FLG expression [17]. The p38 and ERK pathways are activated and promote keratinocyte differentiation when the epidermal barrier is disrupted [18]. Moreover, ERK1/2 can negatively regulate p38 activity, occasionally suppressing differentiation markers [19].

The skin’s biological clock regulates physiological activities in a circadian manner in response to environmental changes [20–22]. Circadian clock genes, such as PER, cryptochrome (CRY), brain and muscle aryl hydrocarbon receptor nuclear translocator (ARNT)-like protein-1 (BMAL1), and circadian locomotor output cycles kaput (CLOCK), are expressed in skin cells in a phased manner and contribute to skin homeostasis and stress mediation [22–24]. Each skin cell type possesses its own circadian system, which is composed of specific genes [25].

Recent findings indicate that disruptions in circadian rhythms contribute to the development of atopic dermatitis [26,27]. Nocturnal increases in blood flow, decreased sebum production, and histamine release intensify nighttime pruritus [28,29]. Skin barrier properties vary with the circadian rhythm, influencing epidermal water loss, stratum corneum hydration, sebum production, skin surface pH, and temperature. Trans-epidermal water loss (TEWL) peaks occur in the late afternoon and evening [30,31]. CLOCK genes influence the regulation of aquaporin-3 (AQP3) proteins, with deficiencies leading to decreased stratum corneum hydration [24]. The differentiation of keratinocytes, vital for skin barrier formation, is controlled by the circadian clock, which triggers differentiation genes during the late night and early morning hours. PER1 and PER2, predominantly expressed in the early morning, facilitate keratinocyte differentiation [22,32]. Leveraging the 24-hour periodicity of the biological clock, chronotherapy has emerged as a promising approach to enhance therapeutic outcomes by aligning with the body’s natural rhythms [33,34]. Chronotherapy for atopic dermatitis could take into account daily patterns of trans-epidermal water loss (TEWL), hydration, sebum production, chemical absorption, and keratinocyte differentiation.

*Cinnamomum cassia*, widely known as cinnamon, contains derivatives such as cinnamaldehyde, cinnamic acid, cinnamate, and various polyphenols [35]. Cinnamon possesses extensive therapeutic properties, encompassing antioxidant, anti-inflammatory, anti-diabetic, antimicrobial, and anticancer effects. It has proven effective against neurological disorders and various chronic diseases [36]. In Traditional Chinese Medicine, cinnamon is utilized to treat colds, enhance blood circulation, warm the body, and boost kidney and lung function, in addition to alleviating low energy and depression [37–39]. Furthermore, the bark of *Cinnamomum cassia* is employed in skincare due to its beneficial effects like anti-aging and skin whitening [40–42]. Nevertheless, the impacts of CCBE on enhancing the skin barrier and its potential applications in chronotherapy and skincare, particularly in the time-dependent regulation of keratinocyte differentiation, are yet to be fully determined. Our study seeks to investigate these potentials.

Thus, our study seeks to elucidate the skin barrier enhancement properties of CCBE and identify its potential applications. We investigated the effects of CCBE on the circadian rhythm and keratinocyte differentiation, assessing its capacity to fortify the skin barrier through the circadian clock in a time-of-day dependent manner via the MAPK pathways, offering a novel method for skin barrier enhancement.

## 2. Materials and methods

### 2.1. Preparation of CCBE extracts

The bark of *Cinnamomum cassia* was sourced from Samhong Chinese Herbal Medicine Company (South Korea). The bark was dried using a hot air dryer at 35 °C overnight and then ground. Cinnamomum cassia bark (100 g) was dried, pulverized, and extracted with 70% ethanol (1 L) at 75 °C for 3 hours. The resulting extract was filtered through a 5-µm charcoal filter and concentrated by evaporation. The concentrate was dried using a freeze dryer and powdered.

### 2.2. Cell lines, cell culture, and pharmacological treatment

HaCaT (CLS Cell Line Service Gmb H, Germany), a spontaneously immortalized human keratinocyte cell line, and HaCaT reporter cells expressing the *PER2pro*-LUC were maintained in Dulbeccoʼs Modified Eagleʼs Medium (DMEM, Welgene, Korea) supplemented with 10% fetal bovine serum (16000044, FBS, Gibco, USA), 1.8 mM CaCl_2_, and 1% penicillin/streptomycin (15140122, Invitrogen, USA) at 37 °C under 5% CO_2_. HaCaT reporter cells expressing *PER2pro*-LUC were established in our previous study [43].

For CCBE and time-dependent CCBE treatments, HaCaT cells were seeded in cell culture plates in DMEM supplemented with 10% FBS. After reaching 80% confluency, cells were treated with CCBE in DMEM containing 1% FBS for the durations specified for each experiment. To analyze the time-dependent effects of CCBE, after reaching 80% confluency, HaCaT cells were initially synchronized with 1 uM dexamethasone (D1756, Sigma-Aldrich, St. Louis, MO, USA) in DMEM for 1 hour. Subsequently, cells were treated with CCBE in DMEM containing 1% FBS for 24 hours at defined ZT time points. The minimum and maximum levels of PER2 at 16 or 24 hours post-synchronization (corresponding to ZT16 or ZT24) were assessed using data from the *PER2pro-LUC* reporter cell line.

### 2.3. Cell viability assay

The effect of CCBE on the viability of HaCaT cells was assessed using the MTT assay. After reaching 80% confluence, cells were treated with CCBE in DMEM containing 1% FBS. Following 24 hours of treatment, cells were incubated with MTT reagent (1 mg/ml in PBS) for 2 hours. The MTT solution was then aspirated from the wells, and the formazan crystals were dissolved in 150 μl of DMSO. The absorbance was measured at a wavelength of 570 nm using an ELISA microplate reader (Bio-Tek Instruments, Winooski, VT, USA).

### 2.4. Small-interfering RNA experiments

All siRNAs targeting the sequences of PER2 and BMAL1 were acquired from Santacruz Biotechnology (Dallas, TX, USA) (sc-36209, sc-38165). A non-targeting 20-25 nt siRNA (sc-37007) served as a negative control to evaluate off-target effects. HaCaT cells were plated in 12-well white cell culture plates for transfection. Upon reaching 60% confluence, cells were transfected with 10 nM siRNA using Lipofectamine RNAiMAX (13778-075, Invitrogen, Carlsbad, CA, USA) and Opti-MEM (31985062, Gibco, Waltham, MA, USA) following the manufacturer’s instructions. Cells were harvested 48 hours post-transfection and analyzed for mRNA expression.

For co-treatment with CCBE and siRNA, cells were washed with PBS 24 hours after transfection and co-treated with CCBE and siPer2 or control-siRNA for 48 hours. The cells were then harvested for transcript level analysis.

### 2.5 . RNA isolation and quantitative real-time RT–PCR

Total RNA was extracted using the TaKaRa MiniBEST Universal RNA Extraction Kit (9767A, Takara Bio, Japan) as per the manufacturer’s instructions. Complementary DNA was synthesized from 1 μg of total mRNA using amfiRivert cDNA synthesis platinum master mix (R5600, GenDEPOT, USA) following the manufacturer’s guidelines. Real-time reverse transcription PCR was conducted using a CronoSTARTM 96 Real-Time PCR system (Takara Bio, Japan) according to the manufacturer’s instructions. Primer sequences are provided in Table 1.

**Table 1:** List of primer sequences.

Gene name	Accession number	Primer sequence
*PUM1*	NM_001101.5	F: CGGTCGTCCTGAGGATAAAA
		R: CGTACGTGAGGCGTGAGTAA
*IVL*	NM_005547.4	F: CCCACAAAGGGAGAAGTATTG
		R: CACTGCGGGTGGTTATTT
*KRT10*	NM_000421.5	F: AGGGGGCAGTTTCGGAGGTG
		R: AAGTAGGAAGCCAGGCGGTCATT
*TGM1*	NM_000359.3	F: ACGACACGCCTTTCATTT
		R: GCCTTCTCCTCCACATAAAC
*FLG*	NM_002016.1	F: CTCAGCATCCCAAGATGGTC
		R: ATCTACCGATTGCTCGTGGT
*CASP14*	NM_012114.2	F: GCCCCTTCTCCAAGATCAGT
		R: AGCACCCTTGTCTGATCCAA
*CAPN1*	NM_005186	F: GCTTCCAGAATGGCTATGC
		R: ACTAGCTTCCCGTCCTTG
*PER2*	NM_022817.2	F: GACTGCAAACCTGGCACTTC R: GTGTCTGAGGGTTCATCACG
*BMAL1*	NM_001030272.3	F: AAGGATGGCTGTTCAGCACATGA
		R: AAAAATCCATCTGCTGCCCTG

### 2.6. Flow cytometry analysis

A BD Accuri™ C6 Plus (BD Biosciences, Franklin Lakes, NJ, USA) was utilized to analyze cells according to the manufacturer’s instructions. Cell size and complexity were assessed using forward (FSC) and side (SSC) light scattering.

### 2.7. Hyaluronic acid enzyme-linked immunosorbent assay (ELISA)

Proteins (200 μg) in supernatants from control, retinoic acid-treated, and CCBE-treated cells were quantified using a Pierce™ BCA Protein Assay Kit (23227, Thermo Fisher Scientific, USA). ELISA was executed with the Hyaluronan DuoSet 4 (DY3614, R&D systems, Minneapolis, Canada) following the manufacturer’s protocols.

### 2.8. Bioluminescence recording

To monitor live-cell bioluminescence, *PER2pro*-LUC reporter cells were seeded onto white 96-well plates (655083, Griner Bio One, Monroe, CA, USA). Upon reaching confluency, cells were synchronized with 1 µM dexamethasone for one hour in DMEM and subsequently treated with CCBE in DMEM containing 1% fetal calf serum for 24 hours. The medium was then replaced with a recording medium consisting of DMEM supplemented with 1% fetal calf serum, 1% penicillin/streptomycin, 10 mM HEPES pH 7.4 (15630080, Gibco, USA), 0.2 mg/mL hygromycin (H0192.0001, DUCHEFA, Netherlands), and 1 mM luciferin (115144-35-9, Goldbio, USA). Bioluminescence was recorded hourly for three days using an Infinite M200 (Tecan, Männedorf, Switzerland). The period and amplitude were determined from detrended rhythms, obtained by subtracting a 24-hour running average from the absolute raw data, and smoothing it with a 2-hour running average. Detrended data were normalized to the baseline luminescence to account for well-to-well differences prior to treatment. Baseline luminescence for each well was defined as the luminescence measured immediately before compound treatment. The detrended data were analyzed using the Fast Fourier Transform Non-linear Least Squares (FFT-NLLS) algorithm, supported by the BioDare2 program (https://biodare2.ed.ac.uk/) [44]. Analysis covered a range of 16 to 72 hours using the linear trend method. Cell numbers were measured via LUNA-II™ (Logos Biosystems, VA, USA).

### 2.9. Western blotting

Cell lysates were prepared using a protein extraction reagent (71009-3, Merck, Germany) containing protease and phosphatase inhibitors (P3100, P3200, GenDEPOT, Baker, TX 77412, USA). Equal amounts of protein (20 μg) were loaded into each lane, separated by SDS-PAGE on 4∼12% Bis-Tris protein gels (NP0321BOX, Thermo Fisher Scientific, Waltham, MA, USA), and subsequently transferred onto polyvinylidene difluoride (PVDF) membranes. Immunoblotting utilized primary antibodies targeting phospho-p38 MAPK (Thr180/Tyr182) (9216, Cell Signaling Technology, Danvers, MA, USA), p38 MAPK (D13E1) (8690, Invitrogen), Phospho-Erk1/2 (Thr202/Tyr204) (9106, Cell Signaling Technology, Danvers, MA, USA), Erk1/2 (9102, Cell Signaling Technology, Danvers, MA, USA), phospho-JNK (Thr183/Tyr185) (4668, Cell Signaling Technology, Danvers, MA, USA), and JNK (9252, Cell Signaling Technology, Danvers, MA, USA).

### 2.10. High-performance liquid chromatography (HPLC)

Cinnamic acid in CCBE was identified and quantified using a Waters HPLC system (Waters, USA), comprising a Waters 2695 Separation module and a Waters 996 PDA. Separation was achieved via a Phenomenex Luna C18 column (4.6 mm × 250 mm, ID 5 μm), with a sample injection volume of 10 μL. The signal was monitored at 275 nm. The elution gradient started with 10% organic phase B, maintained for 5 minutes, followed by a linear gradient from 10% to 30% over 25 minutes, 30% to 50% over 20 minutes, then 50% to 100% over 2 minutes. It then remained at 100% for 3 minutes before returning to the initial conditions over 2 minutes. Re-equilibration was completed over 13 minutes. The flow rate was maintained at 1.0 mL/min. Each analysis required a total of 70 minutes, including re-equilibration time.

### 2.11. Statistical analysis

Data are presented as mean ± standard error of the mean (SEM) from three replicate measurements and were analyzed one-way ANOVA followed by Dunnett or Bonferroni post hoc. All data conformed to a normal distribution as demonstrated by Levene’s test for homogeneity of variance. Statistical significance was established at *p* < 0.05. All calculations were performed using SPSS software (IBM, Korea).

## 3. Results

### 3.1. CCBE stimulates keratinocyte differentiation

**Fig 1:**
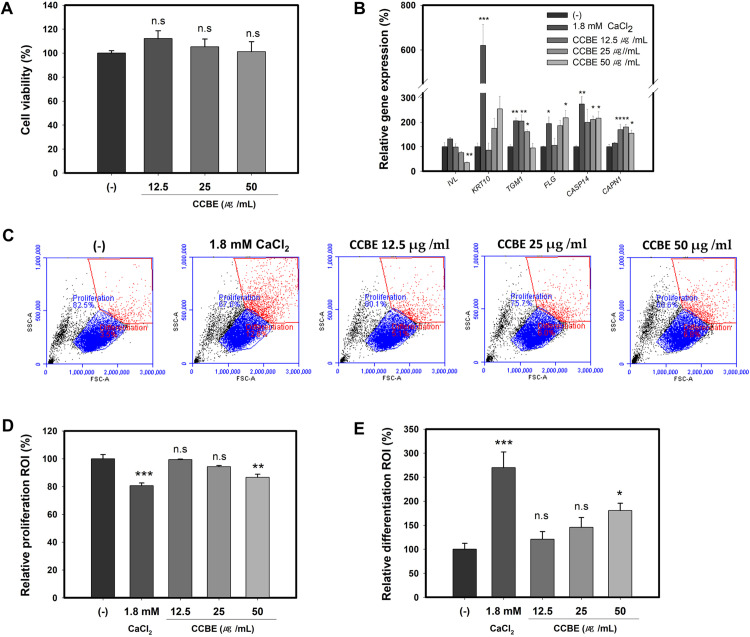
*Cinnamomum cassia* bark extract (CCBE) activates differentiation markers and differentiated populations in HaCaT cells. Cells were treated with (12.5, 25, or 50 μg/mL) CCBE or 1.8mM CaCl2 for 24 hours (A, C-E) or 48 hours (B). The CaCl2-treated groups serve as positive controls for the differentiation of keratinocytes. (A) Cell viability of CCBE. (B) Quantitative PCR analysis details mRNA expression levels of keratinocyte differentiation markers. (C) Flow cytometry identifies basal and differentiated keratinocyte populations. The dot plots display light scattering of front surface area (FSC-A) and side surface area (SSC-A). A blue polygon indicates keratinocytes with low FSC and SSC parameters (basal proliferative keratinocytes), while a red polygon indicates keratinocytes with high FSC and SSC parameters (differentiating keratinocytes). Each region of interest (ROI) indicates the percentage of basal proliferative keratinocytes in the blue polygon (D) or the differentiating keratinocytes in the red polygon (E) relative to the total cell population. Relative values were compared against the control group (-). Data are presented as mean ± standard error of the mean (SEM) based on three replicated measurements (n=3, one-way ANOVA followed by Dunnett post hoc, *P < 0.05, **P < 0.01 compared with the control group (-), n.s, not significant).

We initially examined the cytotoxicity of CCBE on human keratinocyte HaCaT cells. As depicted in Fig 1A, CCBE exhibited no toxicity at concentrations of 12.5, 25, or 50 μg/ml. For subsequent experiments, a concentration of 50 μg/ml was established as the maximum tolerated concentration.

During differentiation, keratinocytes express specific genes in distinct layers of the epidermis. Our research explored the impact of CCBE on the mRNA expression levels of keratinocyte differentiation markers. The differentiation of HaCaT cells can be initiated by a shift from low to high extracellular Ca^2+^ concentration or by achieving a high cell density [45]. To induce the differentiation of HaCaT cells, we cultured the cells at a high density. Upon reaching confluence, the cells were treated with CCBE for 48 hours. As demonstrated in Fig 1B and serving as a positive control, CaCl_2_ significantly induced the expression of keratinocyte differentiation markers, including *KRT10*, *TGM1*, *FLG*, and *CASP14*. Expression levels of *IVL* and *CAPN1* exhibited an increase, although these increases were not statistically significant. CCBE induced mRNA expression of keratinocyte differentiation markers *KRT10*, *FLG*, *CASP14*, and *CAPN1* in a dose-dependent manner. However, the increase in *KRT10* expression was not statistically significant. The expression of *TGM1* was increased at 12.5 and 25 μg/ml CCBE but did not change at 50 μg/ml CCBE. In contrast, the expression of *IVL* decreased at all treatment concentrations.

It is important to note that the expression levels of keratinocyte differentiation markers do not necessarily indicate the degree of differentiation, as each gene exhibits unique expression kinetics. For instance, levels of IVL and some keratin antigens decrease during late differentiation due to cross-linking processes; thus, reduced IVL levels at this stage may signify advanced differentiation rather than a deficiency. Consequently, protein expression levels should be assessed in conjunction with other parameters such as light scattering, which reflects changes in cellular size and complexity [46–49]. As keratinocytes differentiate, they display significant increases in both size and structural complexity, effectively quantifiable through light scattering analysis.

To measure the light scattering associated with keratinocyte differentiation by CCBE, cells were monitored and quantified using flow cytometry. As demonstrated in Fig 1C, cells treated with CaCl_2_ exhibited significant increases in both size and complexity. Cells treated with CCBE showed higher forward scatter (FSC) and side scatter (SSC) parameters, similar to those treated with the positive control, CaCl_2_. Differentiating keratinocytes display heterogeneous and higher FSC and SSC than proliferative cells. As shown in Fig 1D and Fig 1E, CCBE reduced proliferation but increased differentiation populations of keratinocytes in a dose-dependent manner. These results indicate that CCBE effectively stimulates keratinocyte differentiation.

### 3.2. CCBE increases HA levels in keratinocytes

**Fig 2:**
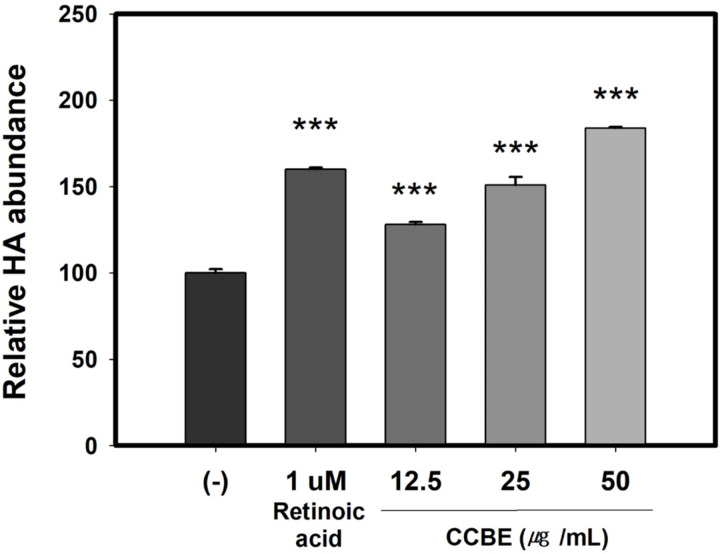
*Cinnamomum cassia* bark extract (CCBE) elevates hyaluronic acid (HA) protein levels in HaCaT cells. Cells were treated with 1 uM retinoic acid or with CCBE at concentrations of 12.5, 25, or 50 μg/mL for 24 hours. HA protein levels were analyzed via enzyme-linked immunosorbent assay (ELISA). Groups treated with retinoic acid served as positive controls. Data are relative values compared with control group (-). They are presented as mean SEM from three replicated measurements (n=3, one-way ANOVA followed by Dunnett post hoc, ***P < 0.001 compared with the control group (-)).

Skin hydration is critical for maintaining a healthy skin barrier, both cosmetically and pathologically. Hyaluronic acid (HA) plays a key role in moisture retention [50,51]. To understand the impact of CCBE on HA production, we treated HaCaT cells with CCBE and measured the HA protein levels. As demonstrated in Fig 2, CCBE positively influenced HA protein levels of keratinocytes in dose-dependent manner.

### 3.3. CCBE enhances PER2 circadian rhythms in keratinocytes

**Fig 3:**
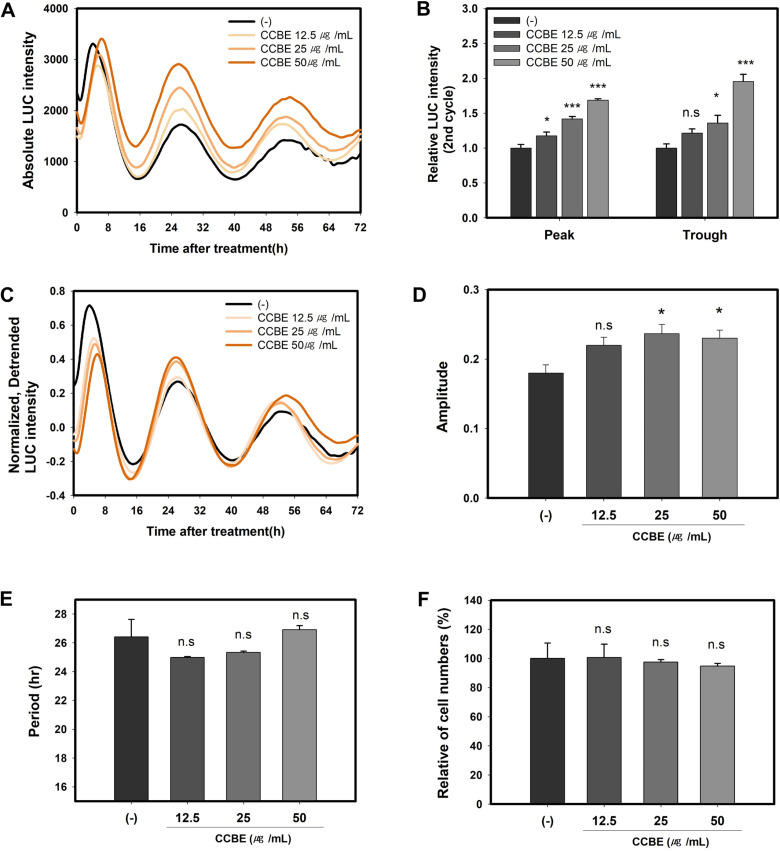
*Cinnamomum cassia* bark extract (CCBE) promotes real-time bioluminescence rhythms in *PER2pro-LUC* reporter cells. Reporter cells were synchronized with dexamethasone and subsequently treated with (12.5, 25, or 50 μg/mL) CCBE for 24 hours. Bioluminescence recordings were taken hourly over three consecutive days. (A) The absolute intensity of PER2pro-LUC bioluminescence was quantified. (B) The graph illustrates the relative LUC intensity compared to the control group (-) using the peak or trough absolute intensity from the second cycle. (C) Detrended data sets were generated by subtracting the 24-h running average from the absolute raw data and subsequently smoothing with a 2-h running average. Detrended data were normalized to the baseline luminescence. (D,E) The average amplitude and period were determined from detrended bioluminescence data using the FFT-NLLS suite provided by the BioDare2 program. (F) The relative rate of number changes in cells treated with CCBE at 27 h post-treatment is presented. Data are shown as mean ± SEM from three replicated measurements (n = 3, one-way ANOVA followed by Dunnett post hoc, *P < 0.05, **P < 0.01, ***P < 0.001, compared with the control group (-), n.s, not significant).

The circadian clock optimizes skin barrier function in response to environmental cues. PER genes are instrumental in the differentiation of keratinocytes [32,52]. To investigate the impact of CCBE on *PER2* circadian rhythms, we administered CCBE to HaCaT reporter cell lines expressing *PER2pro-LUC*. After 24 hours, the culture media were exchanged for recording media, and for the subsequent 72 hours, real-time bioluminescence driven by the *PER2* promoter was monitored. At a 50 μg/mL concentration, CCBE enhanced *PER2* bioluminescence by approximately 69% at the peak and 96% at the trough during the second cycle, reflecting peak and trough expressions (Fig 3A and Fig 3B). Analysis using detrended values revealed that the amplitude of *PER2pro-LUC* circadian rhythms increased with higher doses (Fig 3C and Fig 3D). However, the period remained the same at 50 μg/ml and slightly shortened at doses of 12.5 and 25 μg/ml, though these changes were not statistically significant (Fig 3E).

Taken together, these findings illustrate that CCBE augments *PER2* circadian rhythms in a dose-dependent manner. No significant effects on cell viability were observed at 27 hours post-treatment (Fig 3F), suggesting that the increase in *PER2* circadian rhythms in synchronized HaCaT cells is not attributable to cell proliferation.

### 3.4. CCBE induces keratinocyte differentiation markers and PER2 expression in a time-of-day-dependent manner

It has been reported that undifferentiated primary human keratinocytes (PHKs) are more responsive to differentiation cues such as TGF-β and calcium during late night-morning hours. It has been suggested that an increase in the oscillation amplitude of PER promotes a stronger propensity for differentiation in PHKs as peaks of PER1 and PER2 correlate with a predisposition for differentiation. Overexpression of PER1 or PER2 in PHKs leads to spontaneous differentiation, characterized by a marked decrease in clonogenic capacity and increased expression of differentiation markers, including IVL and LOR [32].

**Fig 4:**
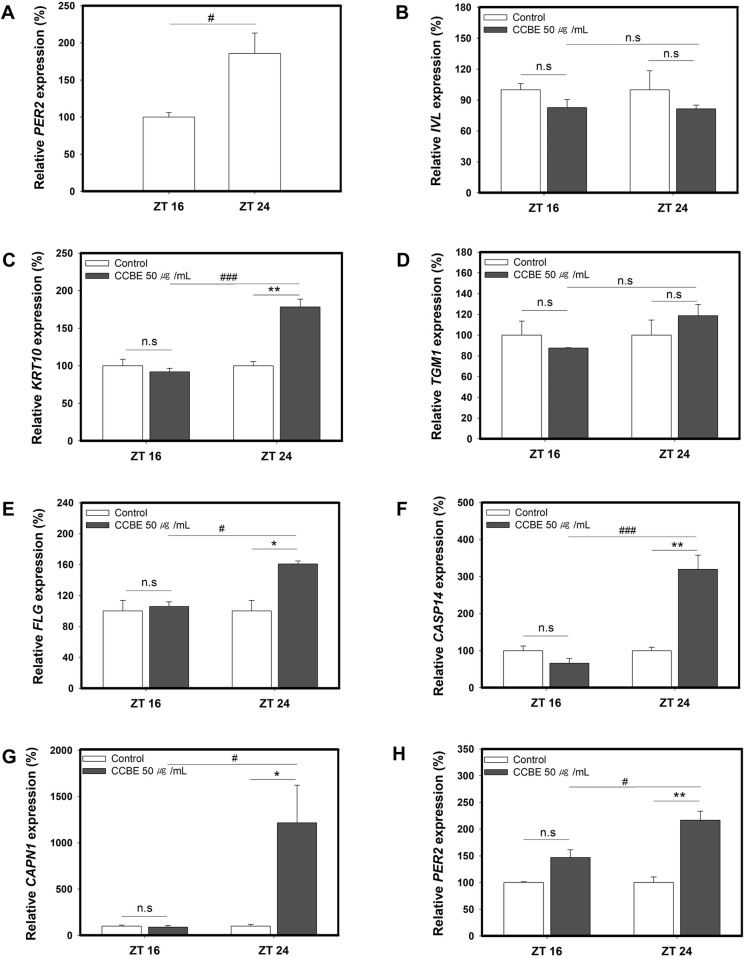
*Cinnamomum cassia* bark extract (CCBE) induces keratinocyte markers and *PER2* predominantly in the morning. Cells were synchronized with dexamethasone and then treated with CCBE for 24 hours at ZT16 and ZT24. (A) A graph displays the relative levels of PER2 expression at ZT16 (evening) and ZT24 (morning) compared to levels at ZT16 (n = 3, two-tailed t-test, *P < 0.05 compared with ZT16). (B-H) Graphs illustrate relative mRNA levels of keratinocyte differentiation markers including IVL(B), KRT10(C), TGM1(D), FLG(E), CASP14(F) and CAPN1(G), and PER2(H). RNA was collected post-CCBE treatment at either ZT 16 or ZT 24. Relative levels were compared with their respective controls 24 hours after each ZT time. Data are represented as mean ± SEM from three replicated measurements (n = 3, one-way ANOVA followed by Bonferroni post hoc, *P < 0.05, **P < 0.01, ***P < 0.001 compared with the control group (-), ^#^P < 0.05, ^###^P < 0.001 compared with ZT 16, n.s, not significant).

We investigated whether CCBE induces differentiation markers and *PER2* in a time-of-day-dependent manner. HaCaT cells were synchronized with dexamethasone and subsequently exposed to CCBE for 24 hours at 16 or 24 hr post-synchronization (ZT 16 and ZT 24), corresponding to evening and morning, respectively. Initially, we verified that *PER2* mRNA expression was higher at ZT 24 (morning) than at ZT 16 (evening) (Fig 4A). We then evaluated the time-responsiveness of cells to CCBE by measuring the expression levels of differentiation markers 24 hours post-CCBE treatment at either ZT16 (evening) or ZT24 (morning). As depicted in Fig 4B–4G, CCBE treatment notably enhanced mRNA expression levels of *KRT10, FLG, CASP14,* and *CAPN1* at ZT 24 (morning) compared with ZT 16 (evening). In particular, *CASP14* and *CAPN1* demonstrated a threefold to twelvefold increase, confirming strong time-dependent responsiveness of *CASP14* and *CAPN1* mRNA expression. Conversely, the mRNA expression levels of *IVL* and *TGM1* did not exhibit significant increases regardless of the ZT time. These findings indicate that CCBE can more effectively induce the expression of specific keratinocyte differentiation markers, including *KRT10*, *FLG*, *CASP14,* and *CAPN1*, in the morning than in the evening. Our data lend support to the hypothesis that the peak of *PER2* is associated with an enhanced receptivity to differentiation cues.

To determine if the morning-specific effect of CCBE was a direct effect mediated through PER2, we examined time-dependent induction of *PER2* expression by CCBE. As demonstrated in Fig 4H, the expression of PER2 induced by CCBE over 24 hours was more efficient at ZT 24 than at ZT 16. This finding aligns with the time-dependent responsiveness of differentiation markers. These results imply that differentiation markers of keratinocytes induced by CCBE are regulated in a morning-specific manner through PER2.

### 3.5. PER2 is essential for CCBE-induced expression of keratinocyte differentiation markers in keratinocytes

We propose that PER2 specifically regulates the morning expression of keratinocyte differentiation markers induced by CCBE. To support this hypothesis, we examined the effects of CCBE on these markers following PER2 knockdown.

**Fig 5:**
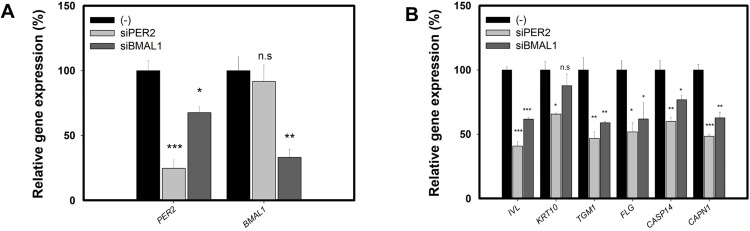
Knockdown of PER2 and BMAL1 diminishes the expression of keratinocyte differentiation markers in HaCaT cells. Cells were transfected with control-siRNA(scrambled), siPER2 and siBMAL1 for 48 hours. (A) A graph displays relative levels of PER2 and BMAL1 expression (B) Graphs illustrate relative mRNA levels of keratinocyte differentiation markers including IVL, KRT10, TGM1, FLG, CASP14 and CAPN1. The relative levels in the siPER2 or siBMAL1 group were compared with those in the control-siRNA groups. Data are presented as mean ± SEM from three replicate measurements (n = 3, one-way ANOVA followed by Dunnett post hoc, *P < 0.05, **P < 0.01, ***P < 0.001, compared with the control-siRNA group (-), n.s, not significant).

We initially utilized the knockdown system of PER2 and BMAL1, specifically expressed at night, to ascertain the role of PER2 and BMAL1 in keratinocyte differentiation markers. As demonstrated in Fig 5A, the endogenous levels of *PER2* and *BMAL1* were effectively reduced by approximately 76% with PER2 siRNA and 67% with BMAL1 siRNA, compared to the scrambled nontargeting siRNA. In Fig 5B, expression levels of the keratinocyte differentiation markers *IVL*, *KRT10*, *TGM1*, *FLG*, *CASP14*, and *CAPN1* were significantly lower in the siPER2 group than in the control-siRNA group. The knockdown of BMAL1 also suppressed the expression of keratinocyte differentiation markers, *IVL*, *TGM1*, *FLG*, *CASP14* and *CAPN1*, but to a lesser degree than the siPER2 group. These findings indicate that both PER2 and BMAL1 influence the expression of keratinocyte differentiation markers. However, BMAL1 is recognized as a transcriptional activator of PER2 [22]. Fig 5A further revealed that knockdown of PER2 specifically reduced the expression of *PER2* but did not affect *BMAL1*, while knockdown of BMAL1 not only diminished the expression of *BMAL1* but also decreased *PER2* expression by about 33%. These results suggest the PER2 knockdown has a direct effect on PER2, whereas the effect of BMAL1 knockdown may be a dual action on keratinocyte differentiation markers via both PER2 and BMAL1.

We examined the effect of CCBE on *BMAL1* expression to determine if there is an indirect influence of BMAL1 on the morning-specific activation by CCBE of keratinocyte differentiation markers. We discovered that CCBE elevated the expression of *PER2* but not *BMAL1* (S1 Fig). These findings suggest that CCBE functions through PER2 rather than BMAL1, although the potential post-transcriptional or translational regulation of BMAL1 by CCBE remains unclear.

**Fig 6:**
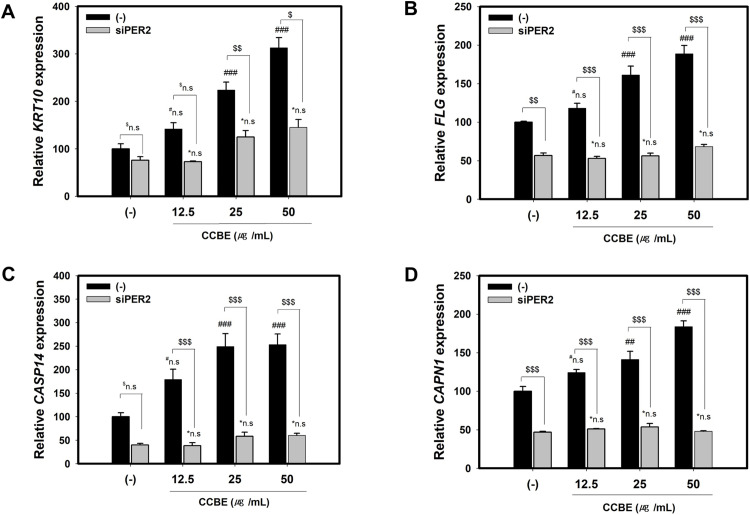
PER2 knockdown diminishes *Cinnamomum cassia* bark extract (CCBE)-induced expressions of keratinocyte differentiation markers *KRT10*, *FLG*, *CASP14* and *CAPN1* in HaCaT cells. Cells were transfected with control siRNA(scrambled) and siPER2 for 24 hours, followed by co-treatment with CCBE (12.5, 25, or 50 μg/mL) and siRNA or siPER2 for 48 hours. (A-D) Graphs display the relative mRNA levels of keratinocyte differentiation markers including KRT10 (A), FLG (B), CASP14 (C), and CAPN1 (D). Relative levels were compared with the control groups (Control-siRNA without CCBE). Data are presented as mean ± SEM from three replicated measurements (n = 3, one-way ANOVA followed by Bonferroni post hoc, ^##^P < 0.01, ^###^P < 0.001, ^#^n.s.; not significant, compared with the control-siRNA(-), *n.s.; not significant compared with the siPER2 group, ^$^P < 0.05, ^$$^P < 0.01, ^$$4^P < 0.001, ^$^n.s.; not significant, compared with the control-siRNA(-) at the same concentration of CCBE).

We then examined the effects of PER2 knockdown on the CCBE-induced keratinocyte differentiation markers, *KRT10*, *FLG*, *CASP14* and *CAPN1*, which previously showed a time-dependent response to CCBE (Fig 4). As demonstrated in Fig 6A–6D, PER2 silencing partially reduced the influence of CCBE on the promotion of keratinocyte differentiation markers, *KRT10*, *FLG*, *CASP14* and *CAPN1* mRNA levels compared to control-siRNA treatment. These results support the conclusion that CCBE-induced keratinocyte differentiation is subject to morning-specific regulation by PER2.

### 3.6. Positive effect of CCBE on the expression of FLG and PER2 is mediated by p38 MAPK signaling

**Fig 7:**
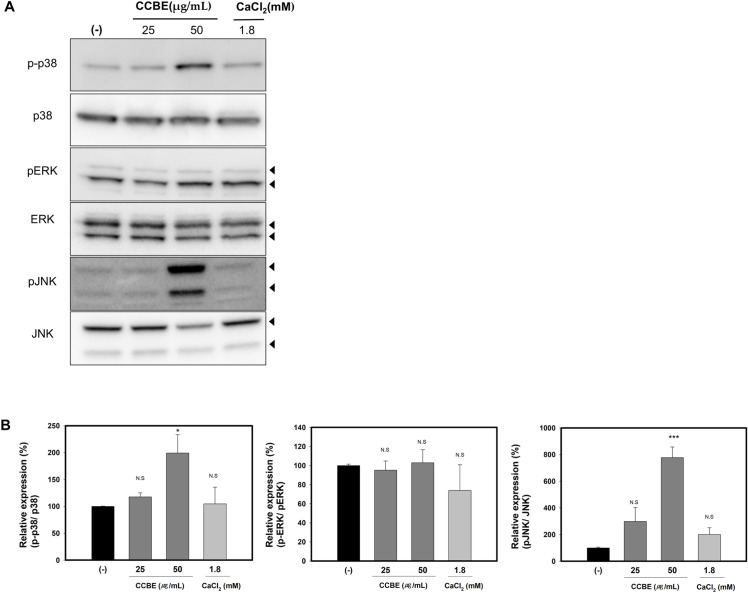
*Cinnamomum cassia* bark extract (CCBE) enhances phosphorylation of p38 and JNK, but not intact proteins. Cells were treated with (25 or 50 μg/mL) CCBE for 30 minutes and then subjected to Western blot analysis using anti-p-p38, p38, p-ERK, ERK, pJNK, and JNK. CaCl2-treated groups served as positive controls. (A) Representative western blots. Blots were probed with antibodies targeting phosphorylated forms of p38, ERK, and JNK. Membranes were stripped and re-probed with antibodies against the total proteins p38, ERK, and JNK, respectively. (B) Densitometry values for phosphorylated proteins relative to total protein. Relative values were compared with the control group (-). Data are presented as mean ± standard error of the mean (SEM) from three replicated measurements (n=3, one-way ANOVA followed by Dunnett post hoc, *P < 0.05, **P < 0.01 compared with the control group (-), n.s, not significant).

To investigate the molecular mechanism of CCBE, we examined its effect on MAPK signaling, specifically p38, ERK, and JNK. The MAPK pathway plays a crucial role in regulating keratinocyte differentiation and maintaining skin barrier function [14,17]. CaCl_2_, serving as a positive control, is known to promote differentiation by activating MAPK signaling pathways, including p38, ERK, and JNK [53]. In the preliminary experiment, we observed that CaCl_2_ rapidly increases the phosphorylation of p38, ERK, and JNK within the first 5 seconds, with effects diminishing after 30 minutes. As depicted in Fig 7A and Fig 7B, CCBE significantly elevates the phosphorylation of p38 and JNK, but does not affect the intact p38 or JNK proteins. CCBE does not influence the levels of phosphorylation or intact ERK proteins. While CaCl_2_ enhances differentiation through the p38, ERK, and JNK MAP kinase pathways, CCBE specifically promotes differentiation through p38 and JNK signaling.

**Fig 8:**
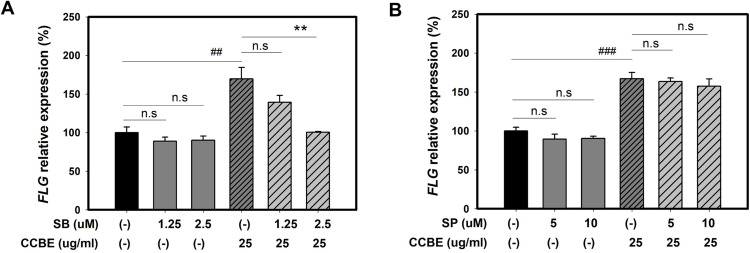
*Cinnamomum cassia* bark extract (CCBE)-induced *FLG* expression is attenuated by the p38 MAPK inhibitor. HaCaT cells were pretreated with (1.25 or 2.5 μM) SB 203580 (A) or (5 or 10 μM) SP600125 (B) for 30 minutes and then co-treated with 25 μg/mL CCBE for 48 hours. Graphs depict relative FLG mRNA expression levels compared to the control group (-) or the CCBE single treated group. Data are presented as mean ± SEM from three replicated measurements (n = 3, one-way ANOVA followed by Bonferroni post hoc, ^##^P< 0.01, ^###^P< 0.001 compared with control group (-), **P < 0.01 compared with CCBE single treated group, n.s, not significant).

We aimed to determine if the effects of CCBE on keratinocyte differentiation could be reversed by specific inhibitors of p38 MAPK and JNK pathways (SB203580 and SP600125, respectively). Cells were pre-treated with each inhibitor for 30 minutes before CCBE treatment. Each inhibitor was also used alone, without co-treatment with CCBE, to confirm their specificity. As shown in Fig 8A and Fig 8B, SB203580 (a p38 MAPK inhibitor) decreased CCBE-induced *FLG* expression in a dose-dependent manner, though there was no significant reduction with the inhibitor alone. Conversely, SP600125 (JNK inhibitor) did not reduce CCBE-induced *FLG* expression. These findings suggest that the enhancement of *FLG* expression in response to CCBE may be mediated by the activation of the p38 MAPK pathway.

**Fig 9:**
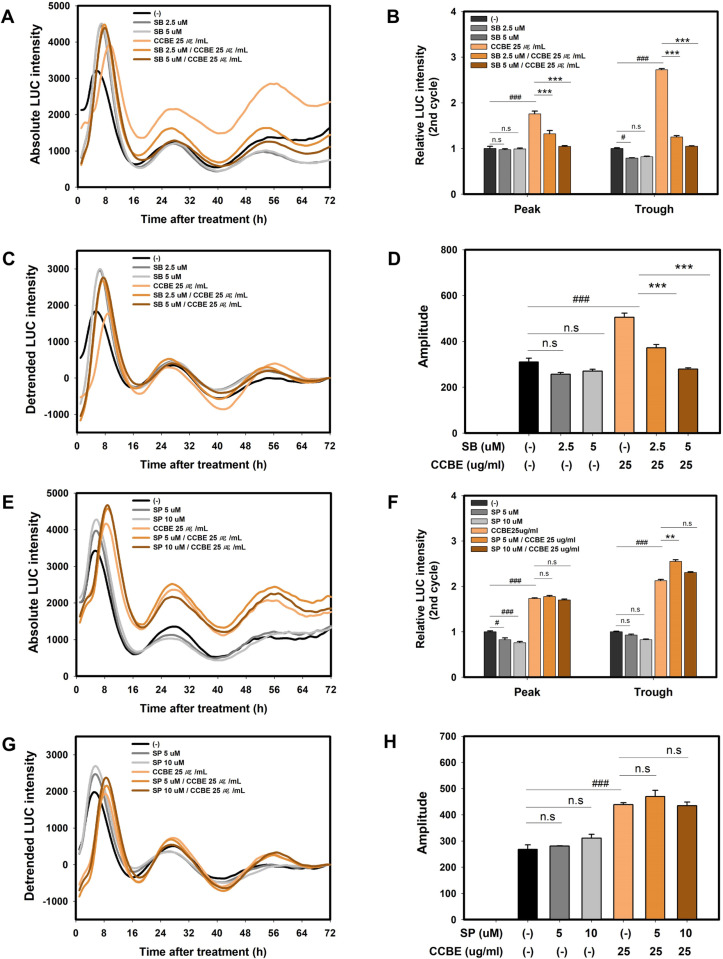
*Cinnamomum cassia* bark extract (CCBE)-induced *PER2* circadian rhythms is inhibited by a p38 MAPK inhibitor. *PER*PER2pro-LUC reporter cell lines were pretreated with (2.5 or 5 μM) SB 203580 (A-D) or (5 or 10 μM) SP600125 (E-H) for 30 minutes, and then co-treated with 25 μg/mL CCBE for 24 hours (A, E). The absolute intensity of PER2pro-LUC bioluminescence was recorded. (B, F) The graph shows the relative LUC intensity compared to the control group (-) or CCBE single treated group, using the peak or trough of the 2nd cycle absolute intensity. (C, G) The graph displays the detrended rhythms. (D, H) Average amplitudes were calculated from the detrended bioluminescence values using the FFT-NLLS suite in the BioDare2 program. Data are presented as mean ± SEM from three replicated measurements (n = 3, one-way ANOVA followed by Bonferroni post hoc, ^#^P< 0.05, ^##^P< 0.01, ^###^P< 0.001 compared with the control group (-), *P < 0.05, **P < 0.01, ***P < 0.001 compared with the CCBE single-treated group, n.s, not significant).

The effects of two inhibitors on CCBE-induced *PER2* expression were measured using a *PER2pro-LUC* reporter cell line. As demonstrated in Fig 9A and Fig 9B, SB203580 (a p38 MAPK inhibitor) dose-dependently reduced CCBE-induced *PER2* bioluminescence at concentrations where neither treatment alone had any significant effect. The increase in amplitude caused by CCBE was also attenuated under conditions treated with the p38 MAPK inhibitor (Fig 9C and Fig 9D). However, when cells were treated with SP600125 (a JNK inhibitor), CCBE still enhanced *PER2* bioluminescence and amplitude (Fig 9E–9H). Collectively, these findings suggest that CCBE could induce *PER2* circadian rhythms and *FLG* expression through p38 MAPK signaling.

### 3.7. Analysis of CCBE

**Fig 10:**
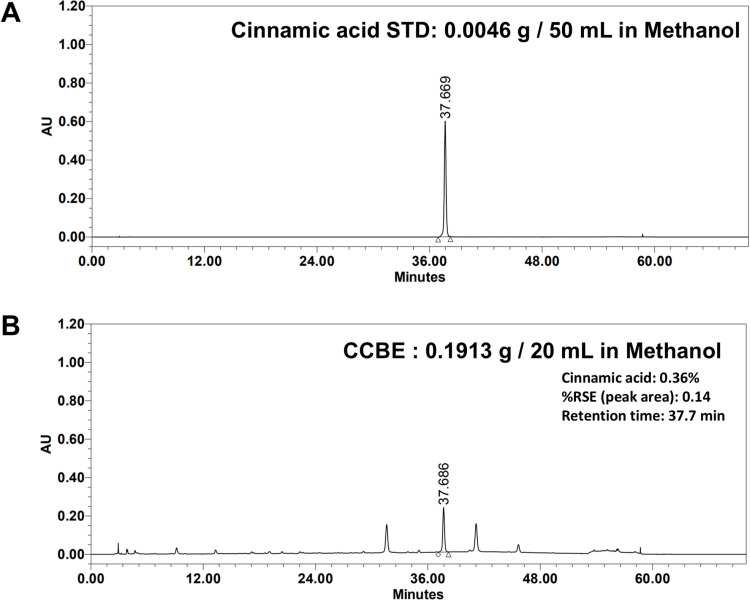
Analysis of *Cinnamomum cassia* bark extract (CCBE). (A) HPLC chromatogram of the cinnamic acid standard (STD) at 275 nm. (B) HPLC chromatogram of CCBE at 275 nm. Content values are presented as mean ± SEM from three replicate measurements.

We found that phenolic acids, predominantly cinnamic acid, composed 0.36% of CCBE components. Chromatograms in Fig 10A and Fig 10B were used to identify peaks by comparing retention times with UV spectra of individual standards.

**Fig 11:**
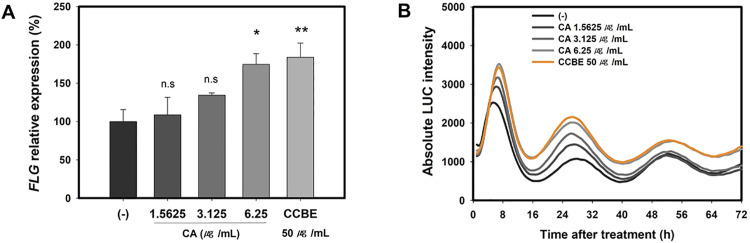
Cinnamic acid enhances *FLG* mRNA expression and circadian rhythms in *PER2pro-LUC* reporter cells. (A) HaCaT cells were treated with various concentrations of cinnamic acid (1.5625, 3.125, or 6.25 μg/mL) for 48 hours. The graphs depict the relative values of FLG mRNA expression compared to the control group (-). (B) PER2pro-LUC reporter cell lines were treated with 1.5625, 3.125, or 6.25 μg/mL cinnamic acid for 24 hours. The absolute intensity of PER2pro-LUC bioluminescence was recorded. Data are presented as mean ± SEM for three replicates (n = 3, one-way ANOVA followed by Dunnett post hoc, *P < 0.05 compared to the control group (-), n.s, not significant).

To elucidate the role of cinnamic acid, we explored its effects on keratinocyte differentiation and circadian clock function. We examined the impact of cinnamic acid on *FLG* mRNA expression in HaCaT cells and the circadian rhythm in *PER2pro-LUC* reporter cells. Fig 11A demonstrates that *FLG* mRNA expression increased by approximately 100% with 6.25 μg/ml cinnamic acid, similar to the increase observed with 50 μg/mL CCBE. According to Fig 11B*, PER2pro-LUC* bioluminescence was enhanced by cinnamic acid in a dose-dependent manner. The effect of 6.25 μg/ml cinnamic acid on *PER2pro-LUC* was comparable to that of 50 μg/mL CCBE. These findings indicate that cinnamic acid is an active component in CCBE that promotes keratinocyte differentiation and enhances circadian clock function.

## 4. Discussion

The skin regulates its functions in response to external environmental cues on a 24-hour cycle through its internal circadian clock, which predicts periodicity and provides functional benefits to the skin. Keratinocytes on the skin’s surface possess a predictive system that actively prepares them to manage external factors such as light [20,54]. The circadian clock genes display peak expression during the day and regulate the cyclic expression of many genes important for skin function [54–58]. In human epidermal stem cells, signals promoting proliferation and differentiation are temporally decoupled [32]. Differentiation genes are expressed in the morning, triggered by calcium and TGF-β. The morning peak of PER gene oscillation is crucial for differentiation propensity. Genes related to UV protection are upregulated during periods of high light exposure. Later, processes such as cell division will peak in the evening when genes for differentiation prepare for the next cycle.

CCBE enhances keratinocyte differentiation markers *KRT10, FLG, CASP14,* and *CAPN1*, particularly in the morning hours. CCBE-induced keratinocyte differentiation markers decrease due to siRNA-mediated PER2 knockdown, suggesting that CCBE may directly regulate the responsiveness of keratinocytes to time-sensitive differentiation signals, instead of merely because cells are more susceptible to these cues in the morning hours. Molecularly, CCBE upregulates *PER2* and *FLG* via p38 MAPK signaling, which is pivotal in regulating keratinocyte differentiation. Though p38 signaling is not a major factor in the photic signaling of PER in the circadian clock, it is crucial for propagating signals from the SCN to peripheral tissues and managing the peripheral clock [59]. The relationship between SCN signaling and the regulation of the skin barrier by the peripheral circadian clock remains to be clarified.

It can be hypothesized that crosstalk between the skin’s biological clock and keratinocyte differentiation occurs via calcium signaling. The calcium gradient across the stratum corneum is crucial for differentiation, with intracellular Ca2 + stores such as the endoplasmic reticulum (ER) and influx mechanisms establishing this gradient. Keratinocyte differentiation genes *KRT10, IVL, TGM1, FLG*, and *CASP14* are upregulated by increased intracellular Ca^2 +^ [60–62]. CAPN1, a FLG-degrading enzyme, is augmented by calcium and is activated upon binding to it [63]. Moreover, the circadian clock influence intracellular Ca^2 +^ regulation, with genes encoding Ca^2 +^ channels and Ca^2 + ^-binding proteins displaying circadian rhythms. Disruption of the circadian clock impact their circadian functionality [64–68]. In the mammalian suprachiasmatic nucleus (SCN), SERCA, a Ca^2 + ^-ATPase uptake system of the endoplasmic reticulum, is expressed nocturnally [65]. The central clock governs extracellular Ca^2 +^ influx through voltage-gated Ca^2+^ channels (VGCCs) [69,70]. Yet, the connection between intracellular Ca^2 +^ and the skin’s circadian clock remain elusive. Only the enhanced expression levels of genes associated with calcium ion homeostasis at the peak time of PER1 and PER2 in human epidermal stem cells have been demonstrated [32]. Consequently, regulating intracellular Ca^2 +^ through the skin’s circadian clock is vital for a deeper understanding of the relation between the skin circadian clock and keratinocyte differentiation. Further research is anticipated into the impact of CCBE on SERCA and VGCCs.

The regulation of aquaporin-3 (*AQP3*) by the CLOCK gene promotes skin hydration via the circadian clock [22,24]. Agerarin from *Ageratum houstonianum* enhances the CLOCK-mediated regulation of the aquaporin-3 (*AQP3*) gene [71]. In this study, we identified CCBE as a novel agent that could strengthen the skin barrier via PER2, a morning circadian clock component. Our results indicate that CCBE induces keratinocyte differentiation and enhances skin barrier function in a time-of-day dependent manner. Keratinocyte differentiation is not simply a cumulative phenomenon over a two-week period but a precisely regulated daily process, highlighting the necessity of meticulously controlling daily moisturizing regimens.

The phytochemistry of Cinnamomum cassia bark has been extensively explored, and the TM-MC database (https://tm-mc.kr/detail.do) indicates it contains 199 constituents. Key components include cinnamaldehyde, cinnamic acid, eugenol, catechins, coumarins, linalool, and beta-caryophyllene. Notably, our study found cinnamic acid as a significant component of CCBE. Cinnamic acid is a natural phenolic acid possessing multiple biological properties such as UV protection, antioxidant activity, antimicrobial effects, skin whitening, hair growth enhancement, and anti-aging properties [72,73]. Additionally, prior studies demonstrate that cinnamic acid can shorten the expression cycle of *PER2pro-LUC* in neuronal cells [74]. In our investigation, cinnamic acid amplified PER2 circadian rhythms (Fig 11B). This variation is likely due to cell type-specific effects. Fig 11A reveals that cinnamic acid increases *FLG* mRNA expression in HaCaT cells. These findings suggest that cinnamic acid is an active compound in CCBE that promotes keratinocyte differentiation and circadian clock function, though further research is essential.

To function as a key promoting compound of CCBE, cinnamic acid must be effective at a concentration of 0.18 μg/mL (0.36% in the extract). Nevertheless, our findings demonstrate that cinnamic acid exerted its effects on FLG and PER2 at a significantly higher concentration of 6.25 μg/mL (Fig 11). This indicates that other active compounds in CCBE might exhibit stronger activity than cinnamic acid, or these compounds may operate synergistically with cinnamic acid. Indeed, our analysis revealed two additional major peaks in CCBE besides cinnamic acid, suggesting multiple components contribute to its biological activity.

## 5. Conclusions

This study demonstrated that *Cinnamomum cassia* bark extract (CCBE) enhances the skin barrier through circadian clock modulation. These findings suggest that CCBE may provide a more effective method for augmenting the skin barrier by specifically targeting keratinocyte differentiation in a time-dependent manner. Additional research is necessary to explore the specific mechanisms through which CCBE influences the circadian clock and keratinocyte differentiation. Furthermore, clinical studies are required to verify the effectiveness of CCBE in treating atopic dermatitis within a chronotherapeutic framework. In conclusion, our research indicates that CCBE holds considerable promise as a novel chronotherapeutic agent, particularly in enhancing skin health and managing atopic dermatitis through the modulation of keratinocyte differentiation according to circadian rhythms.

## Supporting information

S1 Fig CCBE enhances *PER2* but not *BMAL1* expression. Cells were treated with CCBE at doses of 12.5, 25, or 50 μg/mL for 48 hours. A graph displays the relative levels of *PER2* and *BMAL1* expressions. Relative values were compared with the control group (-). Data are presented as mean ±  standard error of the mean (SEM) from three replicated measurements (n = 3, one-way ANOVA followed by Dunnett post hoc, * *P* <  0.05, **P <  0.01 compared with the control group (-), n.s, not significant).
(TIFF)

S2 Fig Raw images of Fig 7. (PDF)

S1 Dataset Dataset for all figures. (XLSX)

## Abbreviations

BMAL1: brain and muscle aryl hydrocarbon receptor nuclear translocator (ARNT)-like protein-1
CAPN1: calpain-1
CASP14: caspase 14
CCBE: Cinnamomum cassia bark extract
FLG: filaggrin
IVL: involucrin
KRT10: keratin 10
PER2: period circadian regulator 2
TGM1: transglutaminase.
