# Supplementary figures and images for: Time-of-day dependent promotion of keratinocyte differentiation by Cinnamomum cassia bark extract through the p38 MAPK Pathway
Source: PLoS One. 2025 Mar 18;20(3):e0318360. doi: 10.1371/journal.pone.0318360 (PMC11918335; doi:10.1371/journal.pone.0318360)

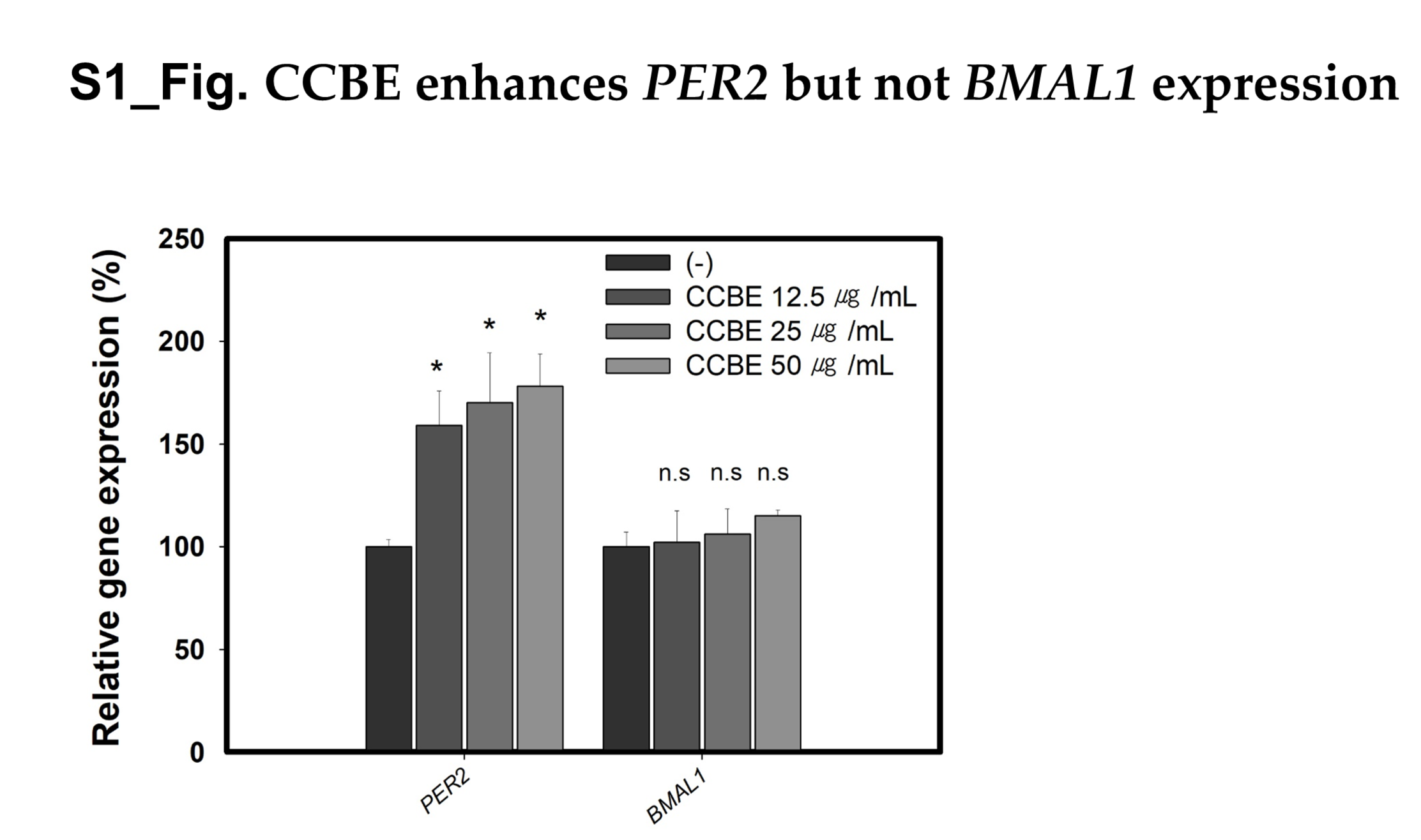

Supplement: S1 Fig — Cells were treated with CCBE at doses of 12.5, 25, or 50 μg/mL for 48 hours. A graph displays the relative levels of PER2 and BMAL1 expressions. Relative values were compared with the control group (-). Data are presented as mean ± standard error of the mean (SEM) from three replicated measurements (n = 3, one-way ANOVA followed by Dunnett post hoc, * P < 0.05, **P < 0.01 compared with the control group (-), n.s, not significant). (TIFF) [file pone.0318360.s001.tiff]

S2 Fig. Raw images in Fig 7

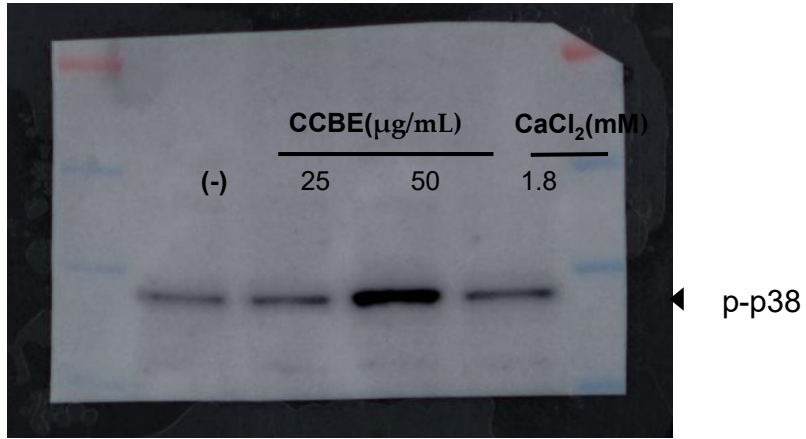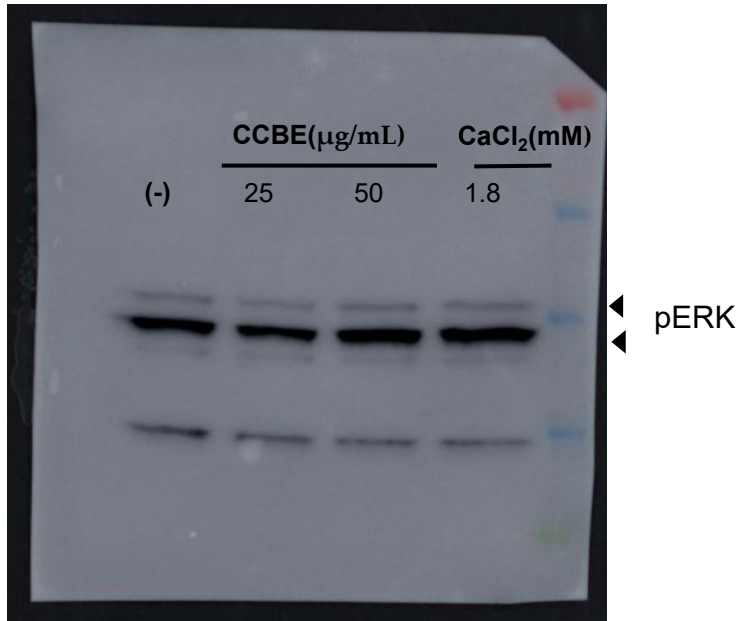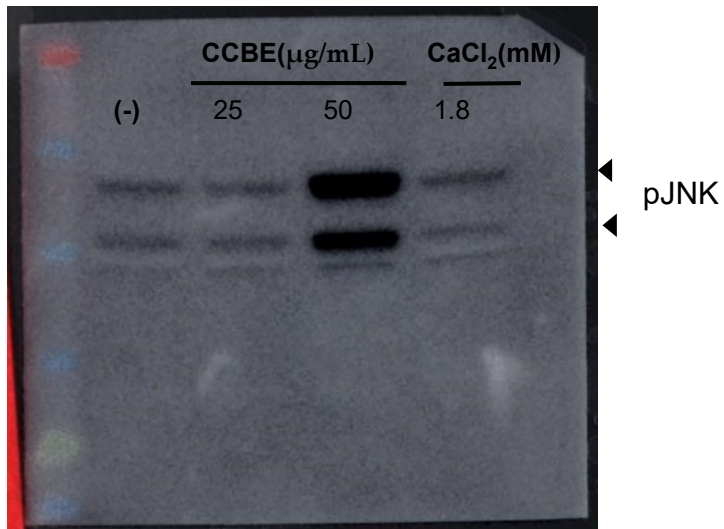

Repeat #1

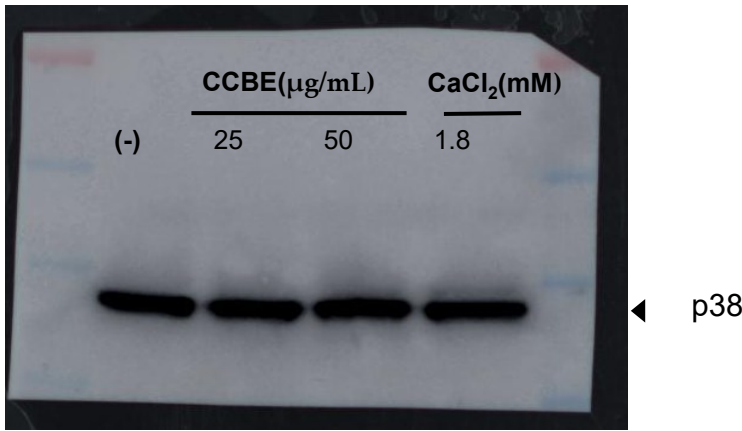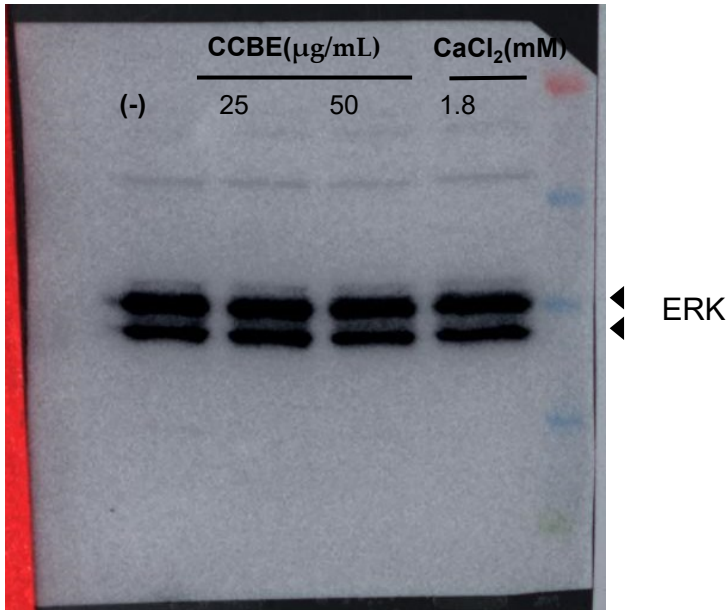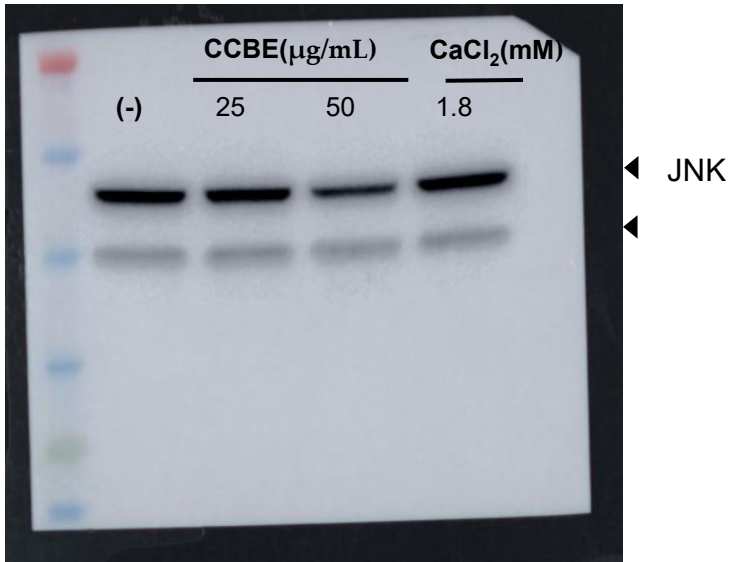

S2 Fig. Raw images in Fig 7

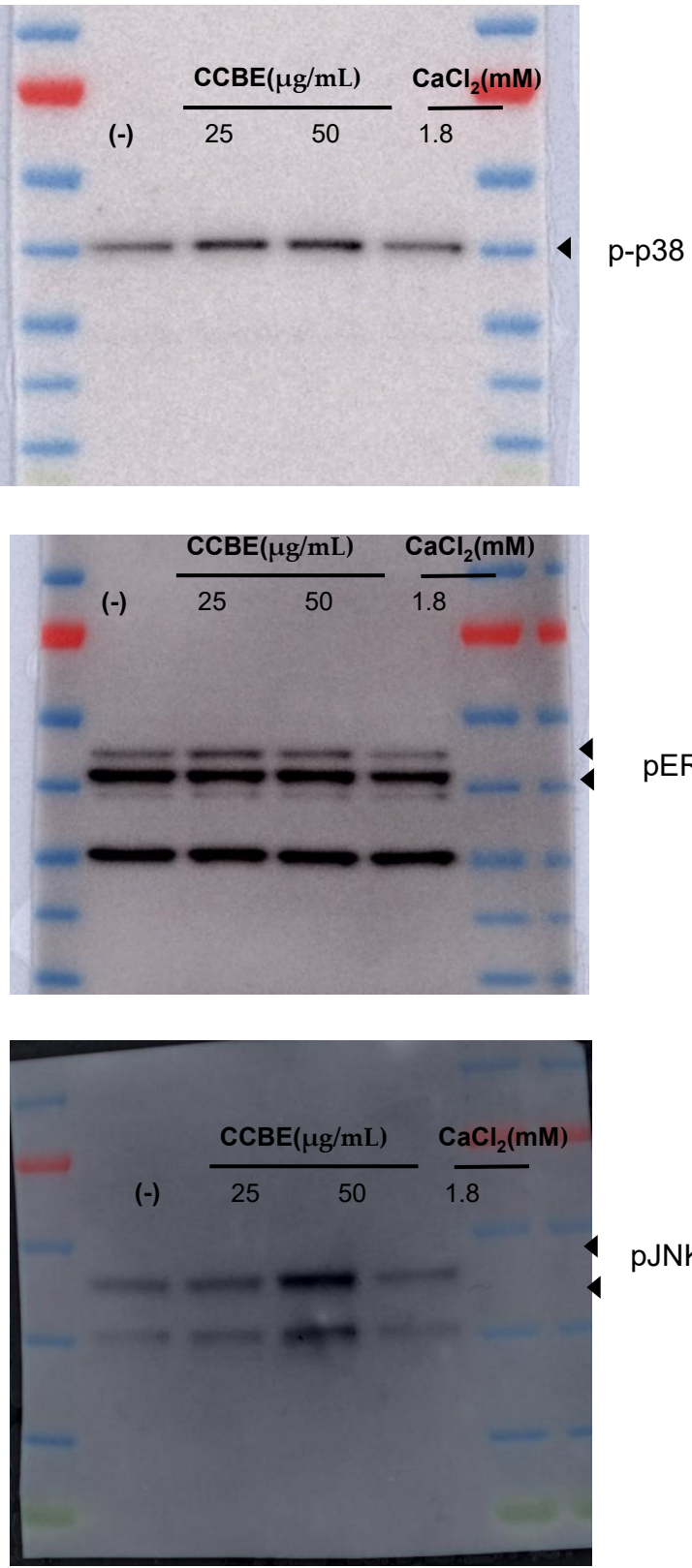

Repeat #2

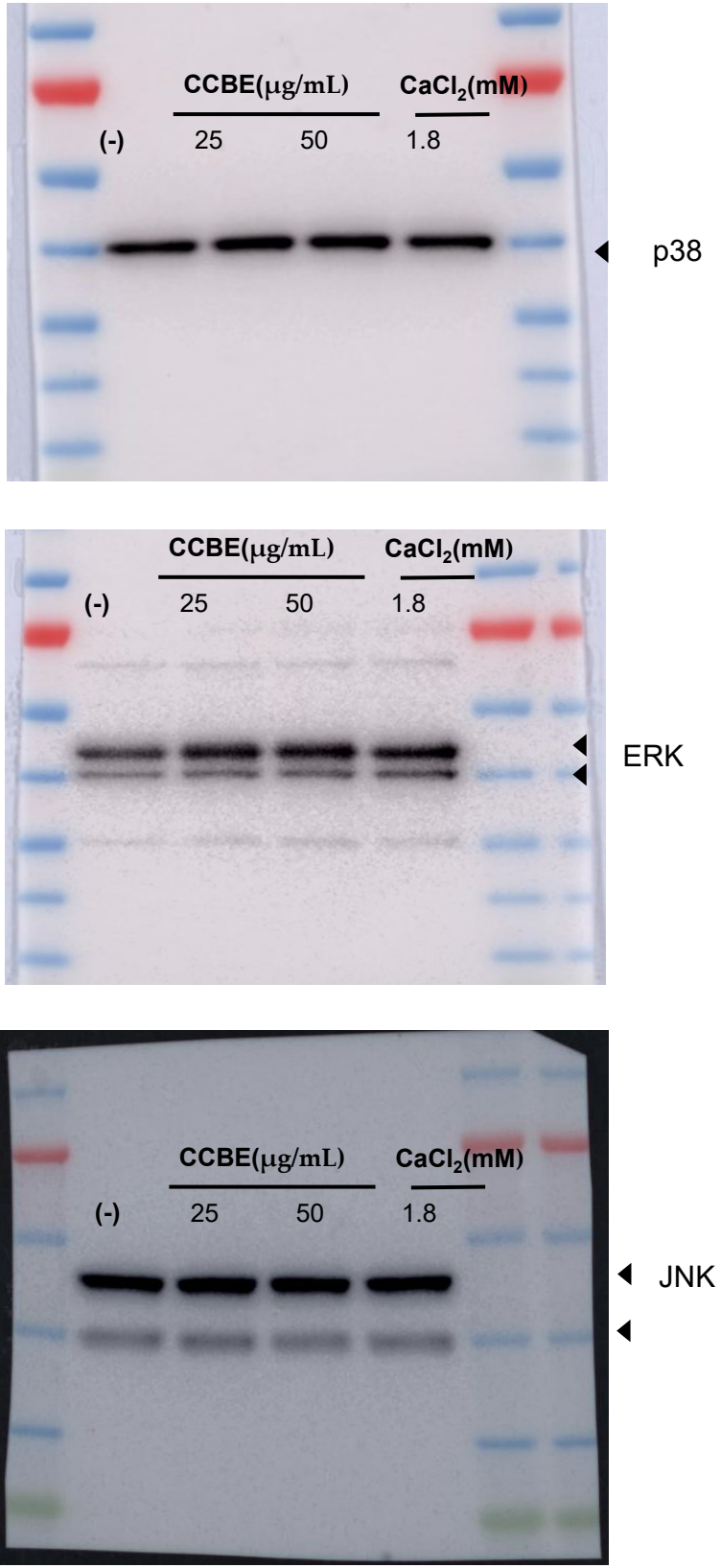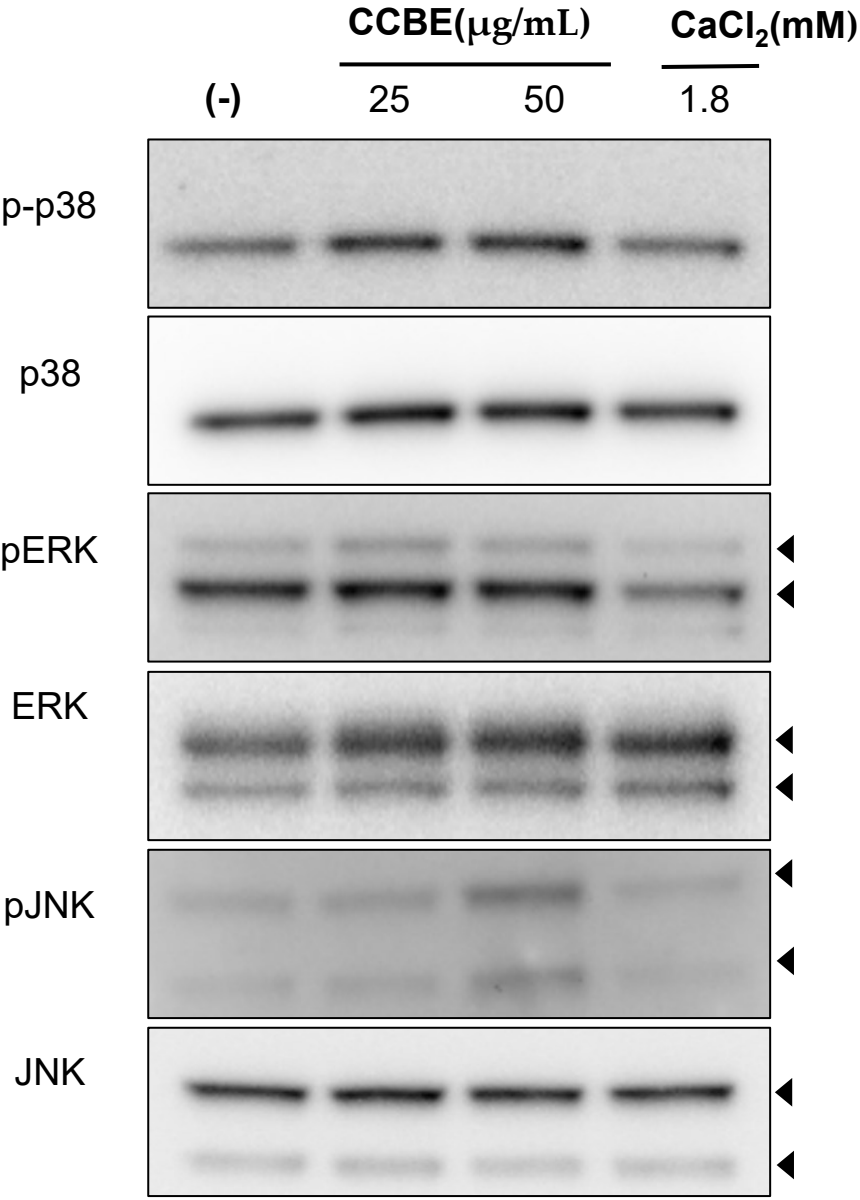

S2 Fig. Raw images in Fig 7

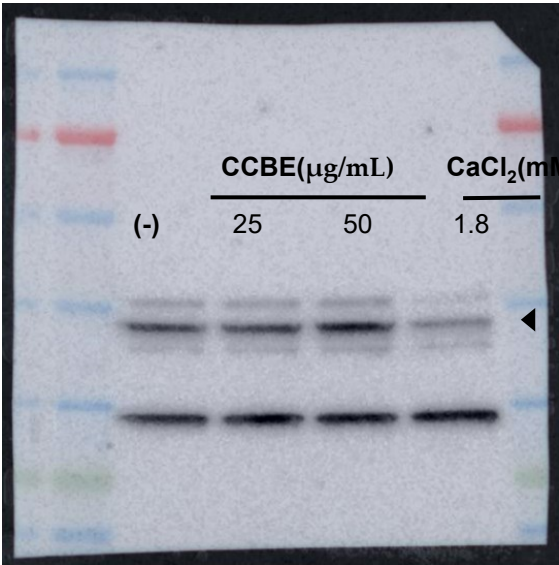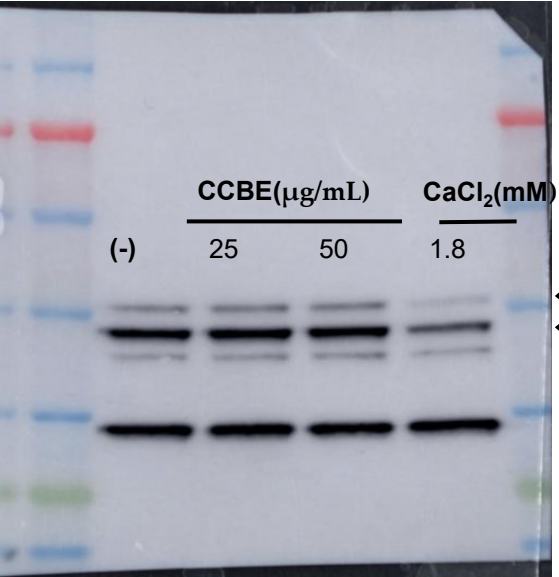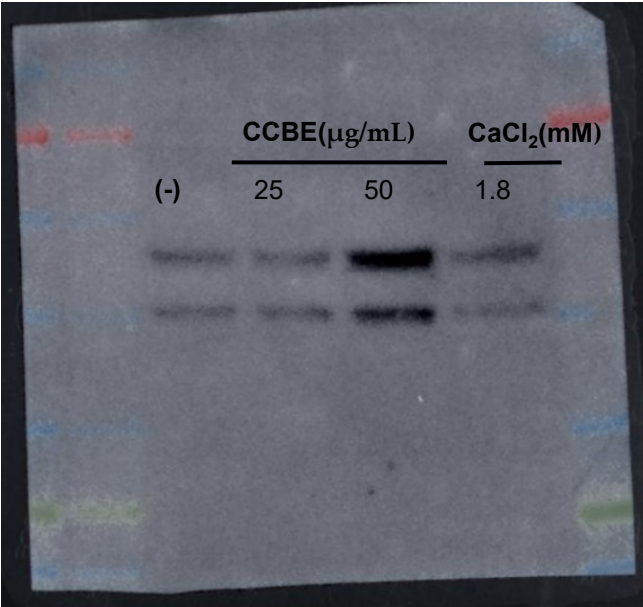

Repeat #3

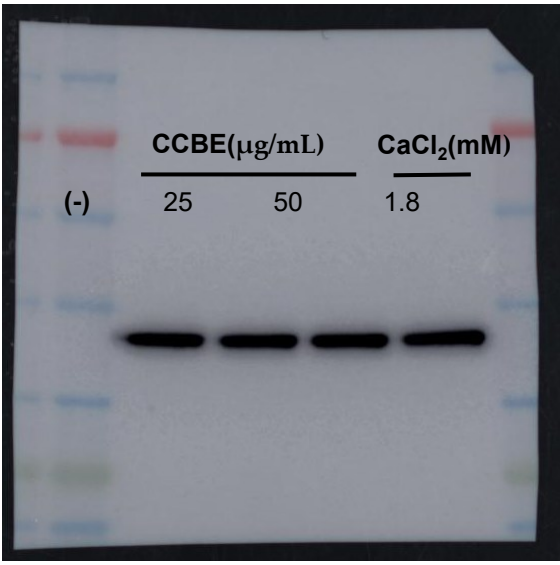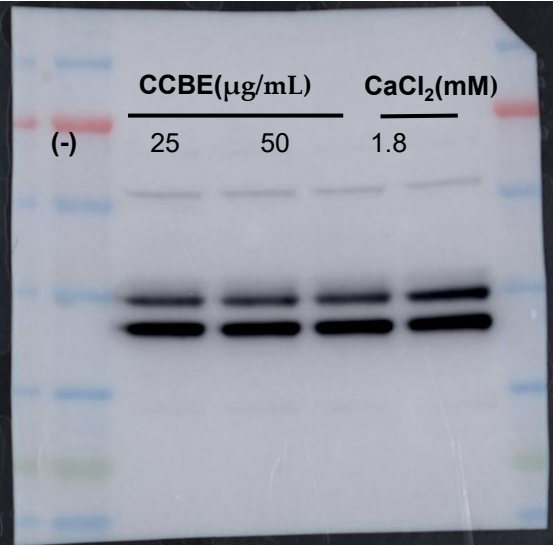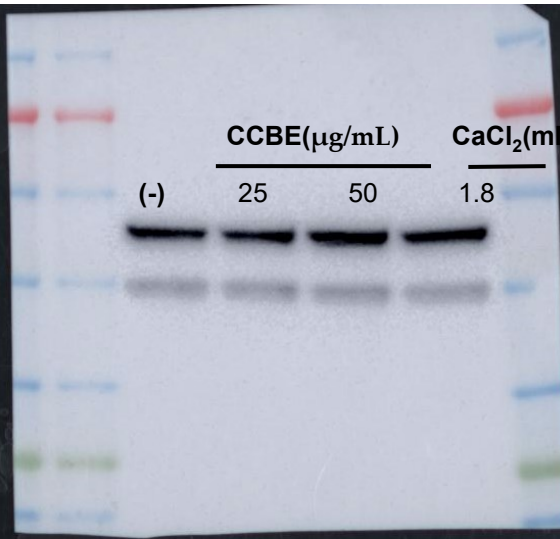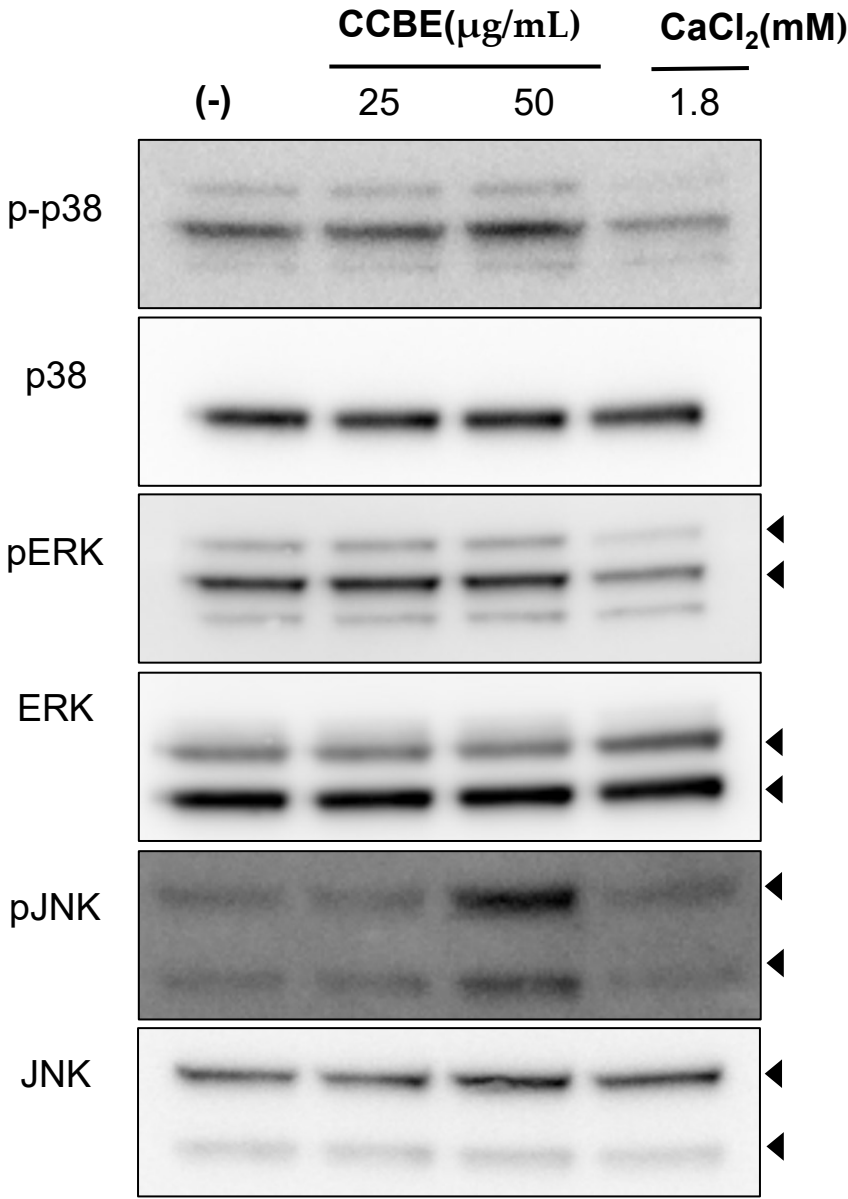

Supplement: S2 Fig — (PDF) [file pone.0318360.s002.pdf]
